# A host-protein signature is superior to other biomarkers for differentiating between bacterial and viral disease in patients with respiratory infection and fever without source: a prospective observational study

**DOI:** 10.1007/s10096-018-3261-3

**Published:** 2018-04-26

**Authors:** Liat Ashkenazi-Hoffnung, Kfir Oved, Roy Navon, Tom Friedman, Olga Boico, Meital Paz, Gali Kronenfeld, Liat Etshtein, Asi Cohen, Tanya M. Gottlieb, Eran Eden, Irina Chistyakov, Isaac Srugo, Adi Klein, Shai Ashkenazi, Oded Scheuerman

**Affiliations:** 1 Department of Pediatrics B, Schneider Children’s Medical Center, Petach Tikva, Israel; 2 Pediatric Infectious Disease Unit, Schneider Children’s Medical Center, Petach Tikva, Israel; 3 Sackler Faculty of Medicine, Tel Aviv University, Tel Aviv, Israel; 4 MeMed, Tirat Carmel, Israel; 5 Rappaport Faculty of Medicine, Technion-Israel Institute of Technology, Haifa, Israel; 6 Department of Pediatrics, Bnai-Zion Medical Center, Haifa, Israel; 7 Department of Pediatrics, Hillel Yaffe Medical Center, Hadera, Israel; 8 Department of Pediatrics A, Schneider Children’s Medical Center, 14 Kaplan Street, 49202 Petach Tikva, Israel

**Keywords:** TRAIL, IP-10, CRP, PCT, Host response

## Abstract

Bacterial and viral infections often present with similar symptoms. Etiologic misdiagnosis can alter the trajectory of patient care, including antibiotic overuse. A host-protein signature comprising tumor necrosis factor-related apoptosis-inducing ligand (TRAIL), interferon gamma-induced protein-10 (IP-10), and C-reactive protein (CRP) was validated recently for differentiating bacterial from viral disease. However, a focused head-to-head comparison of its diagnostic performance against other biomarker candidates for this indication was lacking in patients with respiratory infection and fever without source. We compared the signature to other biomarkers and prediction rules using specimens collected prospectively at two secondary medical centers from children and adults. Inclusion criteria included fever > 37.5 °C, symptom duration ≤ 12 days, and presentation with respiratory infection or fever without source. Comparator method was based on expert panel adjudication. Signature and biomarker cutoffs and prediction rules were predefined. Of 493 potentially eligible patients, 314 were assigned unanimous expert panel diagnosis and also had sufficient specimen volume. The resulting cohort comprised 175 (56%) viral and 139 (44%) bacterial infections. Signature sensitivity 93.5% (95% CI 89.1–97.9%), specificity 94.3% (95% CI 90.7–98.0%), or both were significantly higher (all *p* values < 0.01) than for CRP, procalcitonin, interleukin-6, human neutrophil lipocalin, white blood cell count, absolute neutrophil count, and prediction rules. Signature identified as viral 50/57 viral patients prescribed antibiotics, suggesting potential to reduce antibiotic overuse by 88%. The host-protein signature demonstrated superior diagnostic performance in differentiating viral from bacterial respiratory infections and fever without source. Future utility studies are warranted to validate potential to reduce antibiotic overuse.

**Electronic supplementary material:**

The online version of this article (10.1007/s10096-018-3261-3) contains supplementary material, which is available to authorized users.

## Introduction

Clinicians often encounter the diagnostic challenge of distinguishing between bacterial and viral etiologies in a febrile patient [1]. Medical history, physical findings, and other ancillary medical tests are frequently similar for different causative agents and do not provide definitive discrimination [2, 3]. Misdiagnosis of disease etiology may alter the trajectory of patient care, including over and under use of antibiotics, with fundamental individual and global health consequences.

To aid in accurate clinical decision-making, various laboratory tests are regularly requested [1]. Routine cultures may aid in determining infectious etiology but their utility can be limited by lengthy time to result, low yield, and contamination [4]. Molecular testing expands our capability to detect specific pathogens; yet, test interpretation can be confounded by pathogen co-infections and significant carriage rates of potentially pathogenic microorganisms, such that molecular tests may contribute to over and under diagnosis [5]. Notably, pathogen-based tests are inherently limited by requirement to sample the infection focus, which is especially challenging in lower respiratory infections and fever without source. Therefore, there is pressing need for new reliable and rapid testing to aid the clinician in discriminating between bacterial and viral infections.

Host biomarkers hold great promise as routine diagnostic tools as this approach can overcome many of the previously described challenges [6, 7]. Multiple candidates have been documented, including traditional cellular markers (e.g., white blood cell count, WBC [2] and absolute neutrophil count, ANC [8]) and soluble host-proteins, both classical (e.g., interleukin-6, IL-6 [9]; C-reactive protein, CRP [2, 10–12]; and procalcitonin, PCT [2, 10, 12–14]) and others (e.g., human neutrophil lipocalin, HNL/NGAL [15]). There are also panels of host nucleic acids in the early stages of development [16–18]. Additionally, various prediction models have been proposed that combined several markers, for example, the Lab-score, which integrates PCT, CRP, and urinary dipstick results [19]. To date, wide adoption of such biomarkers and prediction rules for discriminating between bacterial and viral infections has been limited by one or more of the following: lack of rigorous clinical validation [7], narrow applicability (to certain settings, populations, or clinical syndromes) [20], disputed cutoffs [21], and not enough added value beyond standard-of-care [22–24].

Recently, a novel host-protein signature for differentiating between acute bacterial and viral etiologies in children and adults was described [25–29]. This is the first diagnostic test based on soluble host-proteins to include both viral- and bacterial-induced biomarkers: tumor necrosis factor-related apoptosis-inducing ligand (TRAIL), which exhibits induced expression in viral infections and reduced expression in bacterial infection; interferon gamma-induced protein-10 (IP-10) that is induced to a greater extent in viral infections and lesser extent in bacterial infections; and CRP, which exhibits the opposite pattern to IP-10. The diagnostic performance has been validated in two double-blind studies [27, 28], with sensitivity 86.7% (95% confidence interval 75.8–93.1%) and 93.8% (95% CI 87.8–99.8%) and specificity 91.1% (95% CI 87.9–93.6%) and 89.8% (95% CI 85.6–94.0%), respectively. In these former studies, a head-to-head comparison of its diagnostic performance with other biomarkers (at multiple cutoffs) and prediction rules was lacking in patients with respiratory infection (both upper and lower) and fever without source.

In this study, focusing on two prevalent clinical syndromes that are difficult to diagnose as bacterial or viral, we compared the diagnostic performance of the host-protein signature not only to commonly applied CRP and PCT, as documented previously [25, 27, 28, 30], but also in a head-to-head manner to multiple cutoffs of IL-6, HNL, and several prediction rules that have been reported as candidate tools for aiding the clinician in discriminating between bacterial and viral infection.

## Methods

### Study population

Biomarker measurements were performed on specimens from defined subpopulations of the “Curiosity” study that was conducted prospectively at two secondary medical centers in Israel (NCT01917461; Supplementary Materials) [25, 26]. The Curiosity study population comprised inpatients and emergency department (ED) arrivals, both children and adults, presenting with diverse clinical syndromes and a spectrum of pathogens. Inclusion criteria included report of fever > 37.5 °C since onset of symptoms and duration of symptoms ≤ 12 days. Exclusion criteria included: evidence of acute infection in the 2 weeks preceding current presentation; congenital immune deficiency; treatment with immunosuppressive or immunomodulatory agents; active malignancy; and history of human immunodeficiency virus, or hepatitis B/C virus infection. The Curiosity study was approved by the local institutional review boards. Written informed consent was obtained from each participant or legal guardian, as applicable.

The current study included only pediatric and adult patients presenting with one of two clinical syndromes, respiratory infection (upper or lower) and fever without source, and sera available to measure host-protein biomarkers. Diagnosis of respiratory infection required signs or symptoms that involve the upper or lower respiratory tract including the nose, ears, sinuses, pharynx, or larynx as recorded in the electronic case report form. Fever without source required no identified source of infection recorded at presentation after a careful history and a thorough physical examination and a negative urinalysis.

### Data collection

Data on demographics, medical history, physical examination, complete blood count, and chemistry panel were obtained at enrollment. Data were also collected relating to additional diagnostic tests and imaging studies performed on a clinical basis, such as blood culture, throat culture, and serological testing for cytomegalovirus, Epstein-Barr virus, *Mycoplasma pneumonia*e, and *Coxiella burnetii*.

### Specimen analysis

A nasal swab was obtained for microbiological investigation. Nasal swabs were stored at 4 °C for up to 72 h before transport to a central laboratory, where two multiplex polymerase chain reaction analyses were conducted to detect common respiratory viral (Seeplex RV15) and bacterial (Seeplex PB6) pathogens: parainfluenza virus 1, 2, 3, and 4, coronavirus 229E/NL63, adenovirus A/B/C/D/E, bocavirus 1/2/3/4, influenza A, influenza B, metapneumovirus, coronavirus OC43, rhinovirus A/B/C, respiratory syncytial virus A and B, enterovirus, *Streptococcus pneumoniae*, *Haemophilus influenzae*, *Chlamydophila pneumoniae*, *Legionella pneumophila*, *Bordetella pertussis*, and *Mycoplasma pneumoniae*.

A single blood specimen was obtained for measurement of the various biomarkers upon recruitment of the patient to the study; in the case of ED patients, this was at presentation to the ED and in the case of inpatients, it was within 48 h of admission. Venous blood specimens were stored at 4 °C for up to 5 h, subsequently fractionated into serum or plasma and total leukocytes, and stored at − 80 °C. Host-protein biomarkers were measured using the following kits: CRP using either Cobas-6000, Cobas-Integra-400/800, or Modular-Analytics-P800 (Roche); TRAIL and IP-10 using ImmunoXpert™ (MeMed); IL-6 using a commercial ELISA (R&D Systems); PCT using either Elecsys BRAHMS PCT kit or LIAISON BRAHMS PCT kit; HNL using HNL bact ELISA (Diagnostics Development). HNL was measured in the research lab of Schneider Children’s Medical Center on a subset of specimens with sufficient volume (Table 1), with a dilution of 1:100 applied for serum and 1:50 for plasma, according to manufacturer instructions.

**Table 1:** Characteristics of study cohort

Variable	
Study cohort, *n* (%)	
Children, age < 18 years	203 (65)
Adults, age ≥ 18 years	111 (35)
Age in years, mean (SD)	
Children	4.1 (4.0)
Adults	49.8 (19.5)
Gender, *n* (%)	
Male	181 (58)
Female	133 (42)
Received antibiotics, *n* (%)	194 (62)
Maximal temperature in °C, mean (SD)	39.1 (0.75)
Days from symptoms, median (IQR)	3 (2–5)
Presenting signs and symptoms, *n* (%)	
Respiratory	216 (69)
None/fever without a source	98 (31)
Recruitment site, *n* (%)	
Pediatric and adult emergency department	185 (59)
Pediatrics and internal departments	129 (41)
Hospital admission, *n* (%)	189 (61)
Hospitalization duration in days, median (IQR)	2 (0–3)
Site of infection/discharge diagnosis, *n* (%)	
Upper respiratory tract infection^a^	102 (33)
Lower respiratory tract infection^b^	114 (36)
Fever without a source	82 (26)
Bacteremia^c^	12 (4)
Meningitis	2 (0.6)
Lymphadenitis	1 (0.3)
Peritonitis	1 (0.3)

Demographics of the study cohort, *n* = 314. The cohort included only patients with unanimous expert diagnosis; *n*_B_ = 139, *n*_V_ = 175

^a^Included pharyngitis, acute otitis media, aphthous stomatitis, acute sinusitis, and acute tonsillitis

^b^Included pneumonia, bronchiolitis, acute bronchitis, and laryngitis

^c^Included seven cases of septic shock. *n*_B_ = number of patients with unanimous expert panel diagnosis of bacterial infection, *n*_V_ = number of patients with unanimous expert panel diagnosis of viral infection

The CRP, TRAIL and IP-10 measurements, and a small subset of PCT (*n* = 76) and IL-6 (*n* = 43) measurements were performed as part of the Curiosity study [25]. The majority of the PCT (*n* = 238), IL-6 (*n* = 271), and all of the HNL measurements were conducted on frozen serum remnants from the Curiosity study for the purpose of the present study.

Laboratory technicians conducting biomarker tests were blinded to clinical data and comparator method outcomes.

### Index tests

Cutoff values for WBC [2], ANC [8], CRP [2, 10–12], IL-6 [9], and PCT [12–14, 31, 32] were defined prior to data analysis based on literature and guidelines. Due to the lack of established cutoff values for HNL, in addition to applying the previously reported cutoffs for serum [33], we identified and applied additional cutoffs for the current cohort by optimizing for total accuracy for both serum and plasma. Prediction rules combining biomarkers at different cutoffs were defined based on the relevant literature prior to data analysis [19, 34, 35]. The following prediction rules were examined: [CRP < 20 mg/L and PCT < 0.5 ng/mL]; [CRP > 80 mg/L or PCT > 2 ng/mL]; [Lab-score ≥ 3 points] [19]; [CRP ≥ 50 mg/L and PCT ≥ 2 ng/mL and WBC ≥ 15,000/mm^3^] [35]; and [CRP ≥ 30 mg/L or PCT ≥ 0.5 ng/mL or WBC ≥ 15,000/mm^3^] [34]. The Lab-score incorporates PCT and CRP, weighed differently according to their level, and urinary dipstick results: CRP (< 40 mg/L: 0 points; 40–99 mg/L: 2 points; ≥ 100 mg/L: 4 points), PCT (< 0.5 ng/mL: 0 points; ≥ 0.5–1.99 ng/mL: 2 points; ≥ 2.0 ng/mL: 4 points), and positive urine dipstick (1 point).

The host-protein signature score ranging from 0 to 100 is based on computational integration of TRAIL, IP-10, and CRP concentrations and was calculated using the ImmunoXpert™ software (MeMed) [25–27]. Two cutoffs were applied according to manufacturer’s instructions to generate three possible outcomes: (i) viral infection (or other non-bacterial etiology): ImmunoXpert™ score < 35; (ii) equivocal: 35 ≤ ImmunoXpert™ score ≤ 65; and (iii) bacterial infection (including mixed bacterial and viral co-infection): ImmunoXpert™ score > 65. An equivocal outcome is a non-missing, non-erroneous result that does not provide diagnostic information, i.e., is inconclusive. Patients with equivocal outcomes were excluded from analysis of the host-protein signature performance.

### Comparator method

The comparator method applied was expert panel adjudication in line with NHS Health Technology Assessment guidelines for evaluation of diagnostic tests [36]. The panel comprised three independent, experienced, clinically practicing physicians who reviewed after discharge clinical, laboratory, radiological, and microbiological data accrued over the course of the patient’s care, including PCR analysis of nasal swabs. The physicians were blinded to the diagnoses of their peers to prevent group pressure or influential personality bias. Each panel member independently assigned one of the following diagnoses to each patient: (i) bacterial (including mixed bacterial and viral co-infection); (ii) viral; or (iii) indeterminate. The study cohort for the current analysis included only comparator method outcomes when the expert panel were unanimous, i.e., all three panel members independently assigned the same diagnosis. Regarding the biomarkers and prediction rules under study, panel members were provided with CRP, WBC, and ANC data, and blinded to the following: host-protein signature, IL-6, PCT, and HNL, and results of the prediction rules.

### Statistical analysis

Analysis of diagnostic accuracy across the entire cohort was based on total accuracy ((TP + TN)/(P + N)), sensitivity (TP/P), and specificity (TN/N), negative predictive value (NPV = TN/[TN + FN]), and positive predictive value (PPV = TP/[TP + FP]) where P, N, TP, TN, FP, and FN correspond to positives (unanimous expert panel diagnosis bacterial), negatives (unanimous expert panel diagnosis viral), true positives, true negatives, false positives, and false negatives, respectively.

In addition, subgroup analysis based on different age groups, clinical syndromes, pathogens, and microbiological confirmation of unanimous expert diagnosis was performed. Statistical analysis was performed using MATLAB (MathWorks). The *p* values were calculated as follows: for the mean and standard deviation (SD), *t* test; for sensitivity, specificity, and total accuracy, Fisher’s exact test. *p* < 0.05 was deemed statistically significant. *p* values smaller than 0.01 are reported as *p* < 0.01.

## Results

### Patient characteristics

**Fig. 1:**
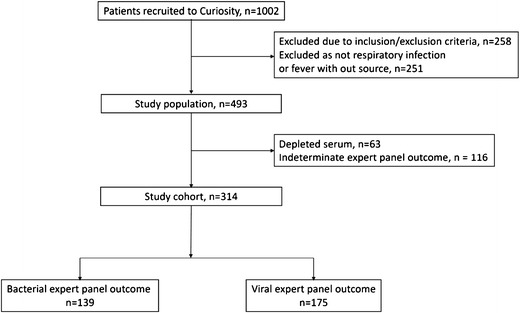
Flow diagram of study population

A total of 493 patients met the inclusion criteria and presented with either respiratory infection (upper or lower) or fever without source. Of these, 430 had adequate serum volume for index test measurements and 314 (73%) were assigned unanimous expert panel diagnoses: 175 (56%) viral and 139 (44%) bacterial (including mixed bacterial and viral) (Fig. 1). The study cohort included 216 patients with a respiratory infection and 98 patients with fever without source (Table 1 and Supplementary Table 1). The ED was the most common recruitment site (59%), with median time from symptom onset to enrollment of 3 days.

Out of the 314 patients assigned unanimous expert panel diagnoses, there were 153 for whom there was clinically relevant microbiological confirmation of the diagnosis, with a total of 23 different organisms detected (Supplementary Table 2). For 16 out of the 98 patients initially presenting with fever without source, a specific clinical diagnosis was recorded at discharge (Table 1), including bacteremia, meningitis, peritonitis, and lymphadenitis. There were no deaths.

### Comparison of host-protein signature to other biomarkers

**Fig. 2:**
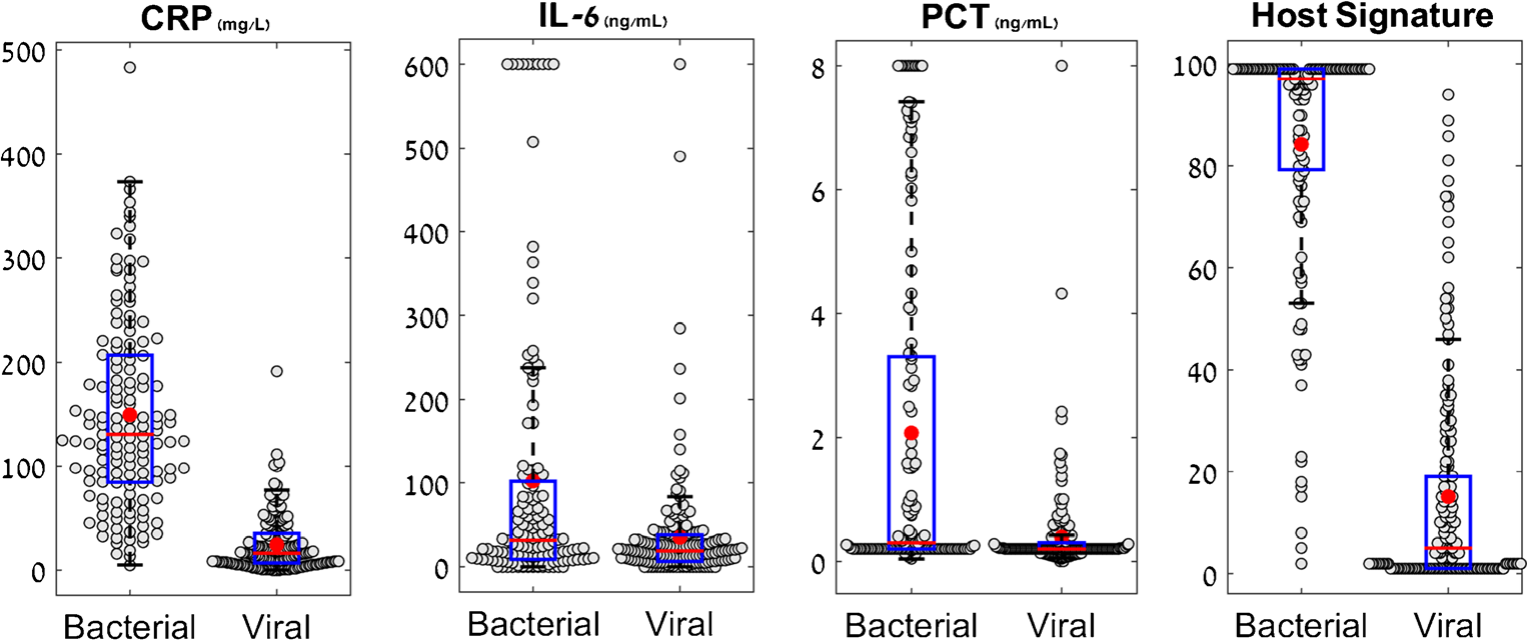
Differential distribution of CRP, IL-6, PCT, and the host-protein signature in bacterial and viral infections. Box plots for CRP, IL-6, PCT, and the host-protein signature measured over the entire study cohort (*n*_B_ = 139, *n*_V_ = 175). The *y*-axis label appears on top of the box plot. Red line corresponds to group median and circle corresponds to group average. The black lines represent the whiskers of the box plot and correspond to 1.5*IQR (interquartile range). *n*_B_ number of patients with unanimous expert panel diagnosis of bacterial infection, *n*_V_ number of patients with unanimous expert panel diagnosis of viral infection

CRP, IL-6, and PCT exhibited higher mean (standard deviation) levels in bacterial as compared to viral infections (Fig. 2; *p* < 0.01): CRP [149 (92) mg/L vs. 25 (27) mg/L]; IL-6 [102 (165) ng/mL vs. 35 (68) ng/mL]; and PCT [2 (3) ng/mL vs. 0.4 (0.7) ng/mL]. The host-protein signature demonstrated the most pronounced differential in bacterial versus viral infections [84 (24) vs. 15 (21), (*p* < 0.01)]. Host-protein signature, CRP, IL-6, and PCT mean levels in mixed infections (bacterial and viral co-infection) are comparable to those found in pure bacterial infections (Supplementary Figure 1; *p* > 0.14).

The host-protein signature yielded significantly higher total accuracy for differentiating between viral and bacterial infections than PCT, CRP, HNL, IL-6, WBC, ANC (all *p* values lower than 0.02; Table 2), while assigning 10.2% of patients equivocal results. The host-protein signature exhibited comparable specificity to PCT at cutoff of 2 ng/mL (*p* = 0.16), yet its sensitivity was significantly higher (93.5% [95% CI 89.1–97.9%] vs. 30.2% [95% CI 22.5–37.9%], *p* < 0.01). Similarly, the host-protein signature demonstrated comparable sensitivity to CRP at cutoff 20 mg/L (*p* = 0.24), but a significantly higher specificity (94.3% [95% CI 90.7–98.0%] vs. 57.1% [95% CI 49.7–64.5%], *p* < 0.01). Notably, despite employing a cutoff optimizing accuracy for HNL of 102.7 μg/L on serum specimens, this protein biomarker yielded significantly lower sensitivity (71.1% [95% CI 55.9–86.2%] vs. 93.5% [95% CI 89.1–97.9%], *p* < 0.01) and specificity (77.5% [95% CI 64.0–91.0%] vs. 94.3% [95% CI 90.7–98.0%], *p* < 0.01) as compared to the host-protein signature.

**Table 2:** Diagnostic performance of host-protein signature compared to biomarkers

Index test	Cutoffs	Total accuracy % (95% CI)	Sensitivity % (95% CI)	Specificity % (95% CI)	PPV % (95% CI)	NPV % (95% CI)
Host-protein signature	< 35 viral > 65 bacterial	94.0 (91.2–96.8)	93.5 (89.1–97.9)	94.3 (90.7–98.0)	92.7 (88.1–97.4)	94.9 (91.3–98.6)
PCT	0.5 ng/mL	65.6 (60.3–70.9)	41.7 (33.4–50.0)	84.6 (79.2–90.0)	68.2 (58.1–78.3)	64.6 (59.9–69.3)
	1 ng/mL	67.8 (62.6–73.0)	36.0 (27.9–44.0)	93.1 (89.4–96.9)	80.6 (70.5–90.8)	64.7 (60.7–68.6)
	2 ng/mL	67.8 (62.6–73.0)	30.2 (22.5–37.9)	97.7 (95.5–100)	91.3 (82.8–99.8)	63.8 (60.2–67.4)
CRP	20 mg/L	74.8 (70.0–79.7)	97.1 (94.3–99.9)	57.1 (49.7–64.5)	64.3 (57.8–70.8)	96.2 (80.2–100)
	40 mg/L	83.4 (79.3–87.6)	90.6 (85.7–95.5)	77.7 (71.5–83.9)	76.4 (69.8–82.9)	91.3 (83.6–99.0)
	80 mg/L	87.6 (83.9–91.2)	77.0 (69.9–84.1)	96.0 (93.1–98.9)	93.9 (89.4–98.3)	84.0 (81.0–87.0)
IL-6	25 pg/mL	57.0 (51.5–62.5)	53.2 (44.8–61.6)	60.0 (52.7–67.3)	51.4 (43.1–59.7)	61.8 (54.2–69.3)
	50 pg/mL	65.0 (59.7–70.3)	40.3 (32.0–48.5)	84.6 (79.2–90.0)	67.5 (57.2–77.8)	64.1 (59.4–68.8)
	100 pg/mL	63.4 (58.0–68.7)	25.2 (17.9–32.5)	93.7 (90.1–97.3)	76.1 (63.3–88.9)	61.2 (57.2–65.1)
HNL (serum)	79 g/L	71.8 (61.6–82.0)	89.5 (79.3–99.7)	55.0 (38.9–71.1)	65.4 (52.0–78.8)	84.6 (55.0–100)
	102.7 g/L	74.4 (64.5–84.3)	71.1 (55.9–86.2)	77.5 (64.0–91.0)	75.0 (60.1–89.9)	73.8 (60.9–86.7)
	167 g/L	69.2 (58.8–79.7)	39.5 (23.2–55.8)	97.5 (92.4–100)	93.8 (80.4–100)	62.9 (55.1–70.7)
HNL (plasma)	41.45 g/L	70.5 (60.2–80.9)	78.9 (65.4–92.5)	62.5 (46.8–78.2)	66.7 (52.3–81.0)	75.8 (55.9–95.7)
WBC	15,000/mm^3^	61.3 (55.8–66.7)	33.3 (25.4–41.3)	83.7 (78.1–89.3)	62.2 (50.8–73.5)	61.0 (56.3–65.8)
	25,000/mm^3^	57.4 (51.9–63.0)	8.0 (3.4–12.5)	97.1 (94.6–99.6)	68.8 (43.2–94.3)	56.8 (53.0–60.6)
ANC	10,000/mm^3^	67.3 (62.1–72.6)	42.0 (33.7–50.4)	87.7 (82.8–92.7)	73.4 (63.5–83.4)	65.2 (60.7–69.7)

For all of the index tests except for HNL, diagnostic performance was evaluated by comparing the expert panel diagnosis (*n*_B_ = 139, *n*_V_ = 175) with the outcome classified by the index test. Predefined cutoffs were applied as indicated. The host-protein signature assigned equivocal results to 10.2% of patients. For HNL, diagnostic performance was evaluated by comparing the expert diagnosis for 78 patients (*n*_B_ = 38, *n*_V_ = 40) with sufficient volume of serum and plasma

*PPV* positive predictive value, *NPV* negative predictive value, *n*_B_ number of patients with unanimous expert panel diagnosis of bacterial infection, *n*_V_ number of patients with unanimous expert panel diagnosis of viral infection

### Comparison of host-protein signature to prediction rules

The host-protein signature yielded significantly improved total accuracy as compared to prediction rules using a combination of PCT and CRP to rule-in (CRP > 80 mg/L or PCT > 2 ng/mL) or rule-out (CRP < 20 mg/L and PCT < 0.5 ng/mL) bacterial infection (*p* < 0.02; Table 3). The host signature exhibited comparable specificity to the rule-in prediction (*p* = 0.99), yet the sensitivity of the host signature was significantly higher (93.5% [95% CI 89.1–97.9%] vs. 81.3% [95% CI 74.7–87.9%], *p* < 0.01). Similarly, the host signature demonstrated comparable sensitivity to the rule-out prediction (*p* = 0.24), but a significantly higher specificity (94.3% [95% CI 90.7–98.0%] vs. 50.9% [95% CI 43.4–58.3%], *p* < 0.01).

**Table 3:** Diagnostic performance of host-protein signature compared to prediction rules

Index test	Total accuracy % (95% CI)	Sensitivity % (95% CI)	Specificity % (95% CI)	PPV % (95% CI)	NPV % (95% CI)
Host-protein signature	94.0 (91.2–96.8)	93.5 (89.1–97.9)	94.3 (90.7–98.0)	92.7 (88.1–97.4)	94.9 (91.3–98.6)
CRP < 20 mg/L and PCT < 0.5 ng/mL	71.3 (66.3–76.4)	97.1 (94.3–99.9)	50.9 (43.4–58.3)	61.1 (54.6–67.6)	95.7 (76.6–100)
CRP > 80 mg/L or PCT > 2 ng/mL	88.5 (85.0–92.1)	81.3 (74.7–87.9)	94.3 (90.8–97.8)	91.9 (87.0–96.8)	86.4 (83.0–89.7)
Lab-score ≥ 3 points^a^	83.1 (79.0–87.3)	73.4 (65.9–80.8)	90.9 (86.5–95.2)	86.4 (80.2–92.7)	81.1 (77.1–85.2)
Thayyil et al. [34]					
CRP ≥ 50 mg/L and PCT ≥ 2 ng/mL and WBC ≥ 15,000/mm^3^	60.6 (55.2–66.1)	11.6 (6.2–17.0)	100 (100–100)	100 (100–100)	58.5 (54.9–62.2)
Olaciregui et al. [35]					
CRP ≥ 30 mg/L or PCT ≥ 0.5 ng/mL or WBC ≥ 15,000/mm^3^	73.9 (69.0–78.8)	97.1 (94.3–99.9)	55.2 (47.7–62.7)	63.5 (57.0–70.1)	96.0 (79.0–100)

For all of the index tests, diagnostic performance was evaluated by comparing the expert panel diagnosis (*n*_B_ = 139, *n*_V_ = 175) with the outcome classified by the index test. Predefined cutoffs were applied as indicated. The host-protein signature assigned equivocal results to 10.2% of patients

*PPV* positive predictive value, *NPV* negative predictive value, *n*_B_ number of patients with unanimous expert panel diagnosis of bacterial infection, *n*_V_ number of patients with unanimous expert panel diagnosis of viral infection

^a^The Lab-score incorporates PCT and CRP, weighed differently according to their level, and urinary dipstick results: CRP (< 40 mg/L: 0 points; 40–99 mg/L: 2 points; ≥ 100 mg/L: 4 points), PCT (< 0.5 ng/mL: 0 points; ≥ 0.5–1.99 ng/mL: 2 points; ≥ 2.0 ng/mL: 4 points), and positive urine dipstick (1 point). [19]

The Lab-score (cutoff ≥ 3) yielded comparable specificity (*p* = 0.30) but significantly reduced sensitivity (73.4% [95% CI 65.9–80.8%] vs. 93.5% [95% CI 89.1–97.9%], *p* < 0.01) as compared to the host-protein signature. The total accuracy of the host-protein signature was significantly superior to the Lab-score for differentiating between bacterial and viral infections (94.0% [95% CI 91.2–96.8%] vs. 83.1% [79.0–87.3%], *p* < 0.01).

Two other prediction rules were examined that combined CRP, PCT, and WBC and, in each case, the total accuracy of the host-protein signature were found to be significantly superior (Table 3; *p* < 0.01). The model proposed by Thayyil et al. [34] of CRP > 50 mg/L, PCT > 2 ng/mL, and WBC > 15,000/mm^3^ yielded significantly improved specificity (100% [CI 100–100%] vs. 94.3% [CI 90.7–98.0%], *p* < 0.01) but significantly lower sensitivity (11.6% [CI 6.2–17.0%] vs. 93.5% [CI 89.1–97.9%], *p* < 0.01) when compared to the host-protein signature. Similarly, the model proposed by Olaciregui et al. [35], CRP > 30 mg/L or PCT > 0.5 ng/mL or WBC > 15,000/mm^3^, yielded comparable sensitivity (*p* = 0.24) but significantly lower specificity than the host signature (55.2% [CI 47.7–62.7%] vs. 94.3% [CI 90.7–98.0%], *p* < 0.01).

### Subgroup analysis of host-protein signature diagnostic performance

**Fig. 3:**
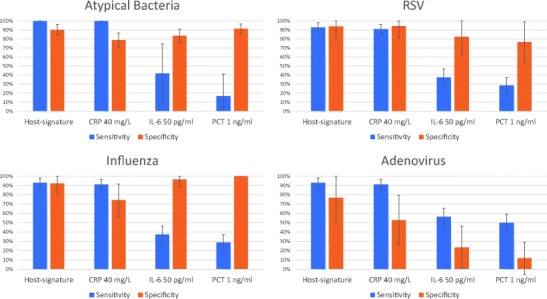
Subgroup analysis of the diagnostic performance of CRP, IL-6, PCT, and the host-protein signature in patients with respiratory infections per pathogen. Diagnostic performance was evaluated by comparing the expert panel diagnosis with the outcome classified by the index test (at the indicated cutoffs) across the subgroup of respiratory infections (*n* = 216, bacterial prevalence = 51.9%) for specific pathogens. Top left panel: Atypical bacterial pathogens, which included *Chlamydophila pneumoniae*, *Mycoplasma pneumoniae*, *Legionella pneumophila* (number of patients with atypical bacterial respiratory infection = 12, number of patients with viral respiratory infection = 104). Top right panel: Respiratory syncytial virus, RSV (number of patients with bacterial respiratory infection = 112, number of patients with RSV infection = 17). Bottom left panel: influenza virus (number of patients with bacterial respiratory infection = 112, number of patients with influenza infection = 27). Bottom right panel: adenovirus (number of patients with bacterial respiratory infection = 112, number of patients with adenovirus = 17). Error bars represent 95% confidence interval. The host-protein signature assigned equivocal results to 10.3, 11.6, 11.5, and 14% of patients for the atypical bacteria, RSV, influenza, and adenovirus subgroups, respectively (see Supplementary Figure 1 for additional index test cutoffs

The performance of the host-protein signature, PCT, CRP, and IL-6 was compared across different subgroups (Tables 4 and 5; Supplementary Table 3; Fig. 3; and Supplementary Figure 2).

**Table 4:** Subgroup analysis in children (age ≤ 18) of the diagnostic performance of the host-protein signature, CRP, IL-6, and PCT

Index test	Cutoffs	Sensitivity % (95% CI)	Specificity % (95% CI)	PPV % (95% CI)	NPV % (95% CI)
Host-protein signature	< 35 viral > 65 bacterial	95.2 (88.5–100)	94.1 (90.1–98.1)	83.3 (72.4–94.3)	98.5 (94.2–100)
PCT	0.5 ng/mL	59.6 (45.8–73.4)	82.8 (76.7–88.9)	54.4 (41.1–67.7)	85.6 (79.3–91.9)
	1 ng/mL	51.9 (37.9–66.0)	92.7 (88.5–96.9)	71.1 (55.9–86.2)	84.8 (80.8–88.9)
	2 ng/mL	50.0 (35.9–64.1)	97.4 (94.8–99.9)	86.7 (73.8–99.6)	85.0 (82.1–87.8)
CRP	20 mg/L	96.2 (90.7–100)	56.3 (48.3–64.3)	43.1 (34.0–52.3)	97.7 (79.5–100)
	40 mg/L	84.6 (74.5–94.8)	77.5 (70.7–84.2)	56.4 (45.2–67.7)	93.6 (84.9–100)
	80 mg/L	63.5 (49.9–77.0)	95.4 (92.0–98.8)	82.5 (70.2–94.8)	88.3 (85.0–91.7)
IL-6	25 pg/mL	59.6 (45.8–73.4)	55.0 (46.9–63.0)	31.3 (22.0–40.6)	79.8 (66.7–92.9)
	50 pg/mL	44.2 (30.3–58.2)	82.1 (75.9–88.3)	46.0 (31.7–60.3)	81.0 (74.9–87.1)
	100 pg/mL	25.0 (12.8–37.2)	92.7 (88.5–96.9)	54.2 (32.7–75.7)	78.2 (74.2–82.3)

Diagnostic performance was evaluated by comparing the comparator method outcome with the outcome classified by the index test, (*n*_B_ = 52, *n*_V_ = 151). The host signature assigned equivocal results to 12.3% of children

*n*_*B*_ number of patients with unanimous expert panel diagnosis of bacterial infection, *n*_*V*_ number of patients with unanimous expert panel diagnosis of viral infection

**Table 5:** Subgroup analysis in adults (age > 18) of the diagnostic performance of the host-protein signature, CRP, IL-6, and PCT

Index test	Cutoffs	Sensitivity % (95% CI)	Specificity % (95% CI)	PPV % (95% CI)	NPV % (95% CI)
Host-protein signature	< 35 viral > 65 bacterial	92.6 (86.8–98.4)	95.7 (86.6–100)	98.7 (96.1–100)	78.6 (69.1–88.1)
PCT	0.5 ng/mL	31.0 (21.1–41.0)	95.8 (87.2–100)	96.4 (89.1–100)	27.7 (19.3–36.1)
	1 ng/mL	26.4 (17.0–35.9)	95.8 (87.2–100)	95.8 (87.2–100)	26.4 (18.3–34.6)
	2 ng/mL	18.4 (10.1–26.7)	100 (100–100)	100 (100–100)	25.3 (17.6–33.0)
CRP	20 mg/L	97.7 (94.5–100)	62.5 (41.6–83.4)	90.4 (84.4–96.5)	88.2 (53.7–100)
	40 mg/L	94.3 (89.3–99.2)	79.2 (61.6–96.7)	94.3 (89.3–99.2)	79.2 (61.6–96.7)
	80 mg/L	85.1 (77.4–92.7)	100 (100–100)	100 (100–100)	64.9 (55.3–74.4)
IL-6	25 pg/mL	49.4 (38.7–60.1)	91.7 (79.7–100)	95.6 (89.3–100)	33.3 (23.7–43.0)
	50 pg/mL	37.9 (27.5–48.3)	100 (100–100)	100 (100–100)	30.8 (22.1–39.5)
	100 pg/mL	25.3 (16.0–34.6)	100 (100–100)	100 (100–100)	27.0 (18.9–35.0)

Diagnostic performance was evaluated by comparing the comparator method outcome with the outcome classified by the index test, (*n*_B_ = 87, *n*_V_ = 24). The host signature assigned equivocal results to 6.3% of adults

*n*_*B*_ number of patients with unanimous expert panel diagnosis of bacterial infection, *n*_*V*_ number of patients with unanimous expert panel diagnosis of viral infection

#### Clinical syndrome: respiratory infections

The diagnostic performance was compared across the subgroup of patients presenting with respiratory infections (number of respiratory patients with unanimous expert panel diagnosis of bacterial infection = 112, number of respiratory patients with unanimous expert panel diagnosis of viral infection = 104). Similar to the findings for the entire cohort, the host-protein signature exhibited superior total accuracy (Supplementary Table 2; *p* < 0.05).

#### Pathogen type

Sensitivity and specificity was examined per pathogen type across the subgroup of patients presenting with respiratory infections (Fig. 3). Out of the 216 patients with respiratory infections, there was microbiological confirmation of atypical bacteria (including *Chlamydophila pneumoniae*, *Mycoplasma pneumoniae*, *Legionella pneumophila*) in 12 patients, and among the viral patients, there was microbiological confirmation of RSV, influenza (A + B) and adenovirus in 17, 27, and 17 patients, respectively. It is notable that in adenovirus infection, the specificity of CRP significantly decreased at all cutoffs (*p* < 0.04), whereas that of the host-protein signature did not (*p* = 0.16) as compared to the specificity attained for all respiratory infections (Supplementary Table 2 and Supplementary Figure 2).

#### Age: adults versus children

The diagnostic performance of the host-protein signature was robust across adults and children (sensitivity 92.6% [95% CI 86.8–98.4%] vs. 95.2% [95% CI 88.5–100%], *p* = 0.71) and specificity (95.7% [95% CI 86.6–100%] vs. 94.1% [95% CI 90.1–98.1%], *p* = 0.99) (Tables 4 and 5). In contrast, PCT displayed significantly decreased sensitivity in adults as compared to children at all cutoffs (*p* < 0.01) and IL-6 exhibited reduced specificity in children versus adults at all cutoffs, with significant reduction at cutoffs of 50 and 250 pg/mL (*p* < 0.03).

### Potential reduction of antibiotic use

Out of the 175 patients unanimously assigned viral by the expert panel, 57 were given antibiotics, indicating a 33% rate of antibiotic overuse. The potential of the host-protein signature to reduce antibiotic use was estimated by considering the following ratio: (number of patients given antibiotics who were assigned viral by unanimous expert diagnosis and viral by host-protein signature, *n* = 50)/(number of patients given antibiotics who were assigned viral by unanimous expert diagnosis, *n* = 57). According to this calculation, the host signature has the potential to reduce unnecessary antibiotic use by 88%. The same estimation performed separately for children and adults, indicates overuse rates of 30 and 46%, respectively, and a potential of the host-protein signature to reduce unnecessary antibiotic use by 87 and 91%, respectively, in these subgroups. Similarly, the same estimation performed separately for hospitalized and non-hospitalized patients, indicated overuse rates of 39 and 28%, respectively, with the potential of the host-protein signature to reduce unnecessary antibiotic use by 78 and 97%, respectively, in each clinical setting.

## Discussion

In this study, we compared the performance of individual and combined biomarkers and prediction rules to a recently developed signature comprising three host-proteins—TRAIL, IP-10, and CRP—in patients with respiratory infections and fever without source. We observed that the host-protein signature yielded significantly superior performance for diagnosing bacterial versus viral infections in these patients as compared to the individual biomarkers CRP, PCT, WBC, ANC, IL-6, and HNL, and their currently used combinations. The signature exhibited the highest total accuracy (94.0% [95% CI 91.2–96.8%]), with sensitivity of 93.5% (95% CI 89.1–97.9%) and specificity of 94.3% (95% CI 90.7–98.0%), the only diagnostic test to show promising utility both for ruling-in and ruling-out bacterial infections. The signature also outperformed five prediction rules, including the Lab-score, and exhibited robust performance across age and various pathogens. In particular, even in cases of adenovirus infection, which can trigger bacterial-like responses leading to misdiagnosis as bacterial infection [37], the host-protein signature maintained performance. This study adds valuable information to previous studies introducing the host-protein signature, in its head-to-head comparison to classical and other (e.g., HNL) biomarkers and to prediction rules, its detailed analysis of performance across specific pathogens and its estimation of the potential for this new diagnostic tool to reduce antibiotic use in two clinical syndromes that are challenging for the clinician to manage.

The biomarkers and prediction rules in this comparative study were selected based either on the breadth of their current clinical use and/or their potential for application in real clinical settings for discriminating between bacterial and viral respiratory infections and fever without source [6, 7]. We did not examine nucleic acid panels as, although several research studies support promising performance for differentiating between bacterial and viral infections [16–18], currently there are no affordable technologies for measuring multiple RNAs in a quantitative manner in under an hour, restricting application of nucleic acid biomarkers at point-of-care. In contrast, proteins are amenable to affordable, user-friendly measurements within minutes, which is essential for broad application of a new test for aiding clinicians to discriminate between bacterial and viral infections. A point-of-care platform for measuring the host-protein signature in 15 min is currently under development.

It is noteworthy that a unique feature of the host-protein signature as compared to the other biomarkers and prediction rules examined in the present study is the inclusion of viral-induced biomarkers. The diagnostic value of integrating bacterial and viral biomarkers is supported by a proof-of-concept study of 54 febrile emergency department patients that reported improved discrimination between microbiologically confirmed bacterial and viral infections when TRAIL, IP-10, and PCT were combined into a model as compared to any of the individual biomarkers [38]. In the case of the signature, it is likely that the distinctive expression dynamics of the three proteins in response to bacterial versus viral infections [25–28] contributes to its superior performance.

A key strength of this study is application of a comparator method based on rigorous expert adjudication. One of the challenges in evaluating tests to distinguish bacterial from viral infection is the lack of a gold standard for etiologic diagnosis [36]. To address this, many studies employ microbiological confirmation as a comparator method, which has the advantage of being well-established and reproducible but the notable disadvantage of restricting the cohort to a small proportion of patients, with likely enrichment for easy to diagnose cases. Indeed, there were 153 patients in the subgroup for whom there was clinically relevant microbiological confirmation of the unanimous expert diagnosis as compared to 314 in the full cohort of patients with unanimous expert diagnosis. Although employing expert panel adjudication has the potential to introduce errors, this comparator method has the fundamental advantage of encompassing a greater proportion of patients and consequently, a study cohort that more closely resembles the real clinical setting, especially relevant for respiratory infections and fever without source that can be hard to diagnose at presentation. Other key strengths are the breadth of index tests compared and that their cutoffs were defined before data analysis.

A limitation of the study is that the study population was restricted to secondary care medical centers. Much antibiotic overuse occurs in outpatient settings, particularly for respiratory infections [39], and since the prevalence of viral infections is typically higher and the severity of disease likely lower, future studies of the signature’s diagnostic performance in physician’s offices are planned. Furthermore, immunocompromised patients and oncology patients were excluded from this study, populations that would benefit greatly from such a test and merit future study. Another limitation is that the expert panelists were provided with CRP, WBC, and ANC data, introducing a potential incorporation bias when evaluating the diagnostic performance of any tests that incorporate one or more of these biomarkers, including the host-protein signature. However, since clinicians often employ CRP, WBC, and ANC as part of routine care to decide the etiology of infection, it was reasoned that the comparator method may be impaired if the panelists were blinded to these data. Notably, despite the largest potential incorporation bias, the individual biomarkers CRP, WBC, and ANC did not yield superior performance.

In conclusion, in this study, the host-protein signature comprising TRAIL, IP-10, and CRP exhibited the highest diagnostic performance for distinguishing between bacterial and viral etiologies in patients with respiratory infections and fever without source. The need for such tests is highlighted by the finding that the treating physicians prescribed antibiotics to one third of the viral-infected patients in the present study cohort. The host-protein signature identified 88% of these cases as viral infections, and therefore, has the potential to reduce antibiotic overuse considerably. Importantly, because both sensitivity and specificity are over 93%, this potential may be fulfilled, as the clinician can be confident about the signature’s performance at both ruling-in and ruling-out bacterial infection. This said, the test is not a substitute for physician education on clinical diagnosis and judicious antibiotic use; the host-protein signature is intended for use in conjunction with clinical assessments and other laboratory findings as an aid to differentiate bacterial from viral infection. Future health and economic outcome research is warranted to evaluate the impact of incorporating the signature into routine patient care.

## Electronic supplementary material

ESM 1 (DOCX 208 kb)
