# Supplementary material for: A host-protein signature is superior to other biomarkers for differentiating between bacterial and viral disease in patients with respiratory infection and fever without source: a prospective observational study
Source: Eur J Clin Microbiol Infect Dis. 2018 Apr 26;37(7):1361–71. doi: 10.1007/s10096-018-3261-3 (PMC6015097; doi:10.1007/s10096-018-3261-3)
Supplement: Supplementary file 1 — (DOCX 208 kb) [file 10096_2018_3261_MOESM1_ESM.docx]

**SUPPLEMENTARY MATERIAL**

**SUPPLEMENTARY TABLES**

Supplementary Table 1: Characteristics of patients with bacterial or viral host-protein signature scores (non-equivocal patients, n = 282) compared to patients with equivocal scores (equivocal patients, n = 32).

| Variable | Equivocal patients n=32 | Non-Equivocal patients n=282 |
| --- | --- | --- |
| Expert diagnosis, n (%) |  |  |
| Bacterial | 16 (50%) | 123 (44%) |
| Viral | 16 (50%) | 159 (56%) |
| Study cohort, n (%) |  |  |
| Children, age<18 years | 25 (78%) | 178 (63%) |
| Adults, age≥18 years | 7 (22%) | 104 (37%) |
| Age in years, mean (SD) |  |  |
| Children | 5.3 (4.7) | 3.9 (3.9) |
| Adults | 53.9 (13.4) | 49.6 (19.8) |
| Gender, n (%) |  |  |
| Male | 16 (50%) | 165 (59%) |
| Female | 16 (50%) | 117 (41%) |
| Received antibiotics, n (%) | 20 (62%) | 174 (62%) |
| Maximal temperature in °C, mean (SD) | 38.9 (0.74) | 39.2 (0.75) |
| Days from symptoms, median (IQR) | 3 (3-5) | 3 (2-5) |
| Presenting signs and symptoms, n (%) |  |  |
| Respiratory | 24 (75%) | 192 (68%) |
| None/fever without a source | 8 (25%) | 90 (32%) |
| Hospital admission, n (%) | 23 (72%) | 166 (59%) |
| Hospitalization duration in days, median (IQR) | 1.5 (0-2.25) | 2 (0-3) |

Supplementary Table 2: List of detected organisms

| 1 | Adenovirus A/B/C/D/E |
| --- | --- |
| 2 | Bocavirus 1/2/3/4 |
| 3 | Cytomegalovirus |
| 4 | Coronavirus 229E/NL63/OC43 |
| 5 | Escherichia coli |
| 6 | Epstein Barr virus |
| 7 | Enterococcus faecalis |
| 8 | Enterovirus |
| 9 | Group A Streptococcus |
| 10 | Group G Streptococcus |
| 11 | Haemophilus influenzae |
| 12 | Influenza A (including H1N1) and B virus |
| 13 | Klebsiella Pneumoniae |
| 14 | Metapneumovirus |
| 15 | Mycoplasma pneumoniae |
| 16 | Parainfluenza virus 1/2/3/4 |
| 17 | Pseudomonas A |
| 18 | Respiratory syncytial virus A/B |
| 19 | Rhinovirus A/B/C |
| 20 | Rotavirus |
| 21 | Staphylococcus aureus |
| 22 | Streptococcus pneumoniae |
| 23 | Varicella zoster virus |

Supplementary Table 3: Subgroup analysis in patients with respiratory infections of the diagnostic performance of the host-protein signature, CRP, IL-6 and PCT.

| Index test | Cutoffs | Total accuracy  % (95% CI) | Sensitivity  % (95% CI) | Specificity  % (95% CI) | PPV  % (95% CI) | NPV  % (95% CI) |
| --- | --- | --- | --- | --- | --- | --- |
| Host-protein signature | <35 viral  >65 bacterial | 91.7  (87.7-95.6) | 92.9  (87.7-98.0) | 90.4  (84.4-96.5) | 91.0  (85.3-96.7) | 92.4  (86.2-98.6) |
| PCT | 0.5 ng/ml | 57.9  (51.2-64.5) | 33.9  (25.0-42.8) | 83.7  (76.4-90.9) | 69.1  (56.5-81.7) | 54.0  (48.1-60.0) |
|  | 1 ng/ ml | 58.8  (52.2-65.4) | 28.6  (20.1-37.1) | 91.3  (85.9-96.8) | 78.0  (64.8-91.3) | 54.3  (48.9-59.7) |
|  | 2 ng/ml | 60.2  (53.6-66.8) | 25.9  (17.7-34.1) | 97.1  (93.8-100) | 90.6  (79.9-100) | 54.9  (49.9-59.9) |
| CRP | 20 mg/L | 75.9  (70.2-81.7) | 97.3  (94.3-100) | 52.9  (43.1-62.6) | 69.0  (61.7-76.3) | 94.8  (71.7-100) |
|  | 40 mg/L | 85.2  (80.4-90.0) | 91.1  (85.7-96.4) | 78.8  (70.9-86.8) | 82.3  (75.4-89.1) | 89.1  (79.8-98.5) |
|  | 80 mg/L | 84.7  (79.9-89.6) | 75.9  (67.8-83.9) | 94.2  (89.7-98.8) | 93.4  (88.2-98.6) | 78.4  (73.8-83.0) |
| IL-6 | 25 pg/ml | 53.7  (47.0-60.4) | 50.0  (40.6-59.4) | 57.7  (48.0-67.3) | 56.0  (46.1-65.9) | 51.7  (43.0-60.4) |
|  | 50 pg/ml | 59.7  (53.1-66.3) | 37.5  (28.4-46.6) | 83.7  (76.4-90.9) | 71.2  (59.3-83.1) | 55.4  (49.4-61.4) |
|  | 100 pg/ml | 55.1  (48.4-61.8) | 20.5  (12.9-28.1) | 92.3  (87.1-97.5) | 74.2  (57.9-90.5) | 51.9  (46.6-57.2) |

Supplementary Table 3 Legend: Diagnostic performance was evaluated by comparing the comparator method outcome with the outcome classified by the index test, (n_B_ = 112, n_V_ = 104). The host-protein signature assigned equivocal results to 11.1% of patients. n_B_ = number of patients with unanimous expert panel diagnosis of bacterial infection; n_V_ = number of patients with unanimous expert panel diagnosis of viral infection_._

**SUPPLEMENTARY FIGURE**

Supplementary Figure 1: Differential distribution of CRP, IL-6, PCT and the host-protein signature in pure bacterial, mixed (bacterial and viral co-infection) and pure viral infections.


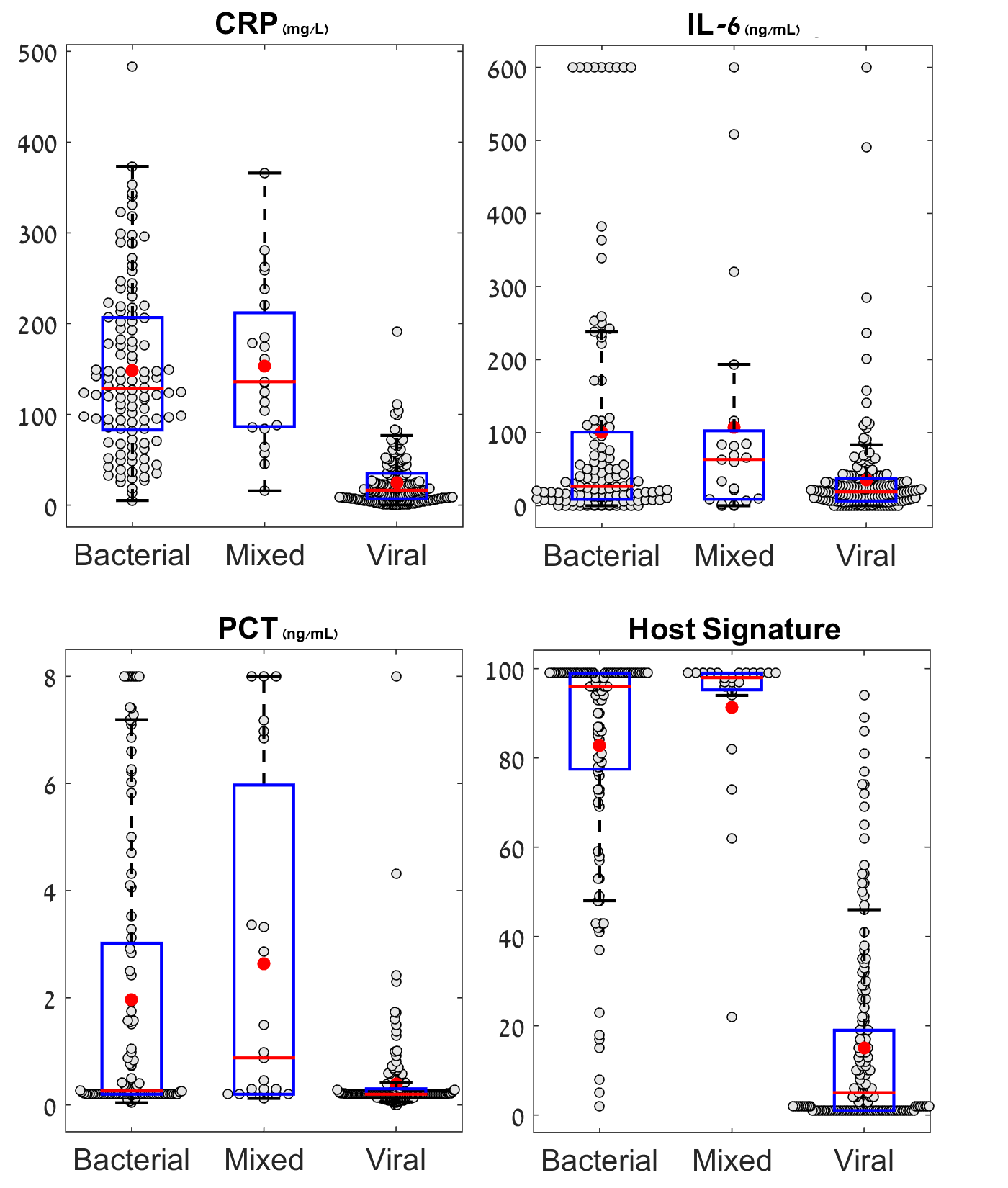


Supplementary Figure 1 Legend: Box plots for CRP, IL-6, PCT and the host-protein signature measured over the entire study cohort (n_B_ = 139, n_V_ = 175). Red line corresponds to group median and circle corresponds to group average. n_B_ = number of patients with unanimous expert panel diagnosis of bacterial infection; n_V_ = number of patients with unanimous expert panel diagnosis of viral infection_._

Supplementary Figure 2: Subgroup analysis of the diagnostic performance of CRP, IL-6, PCT and the host-protein signature in patients with respiratory infections per pathogen.


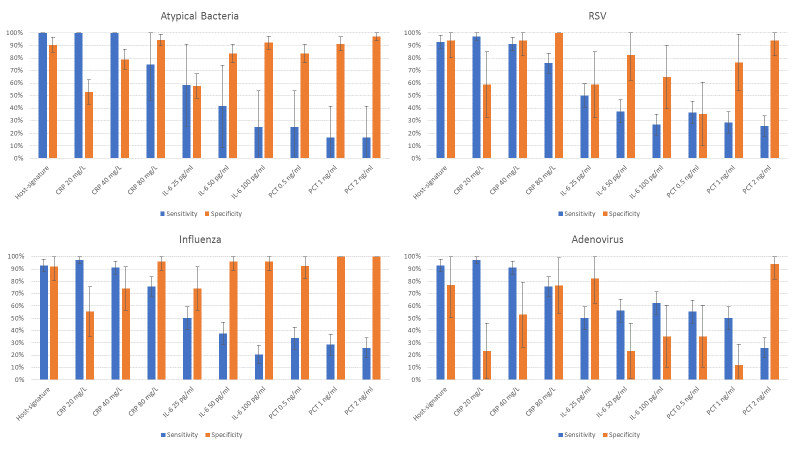


Supplementary Figure 2 Legend: Diagnostic performance was evaluated by comparing the expert panel diagnosis with the outcome classified by the index test (at the indicated cutoffs) across the subgroup of respiratory infections (n=216, bacterial prevalence =51.9%) for specific pathogens.

Top left panel: Atypical bacterial pathogens, which included *Chlamydophila pneumoniae, Mycoplasma pneumoniae, Legionella pneumophila* (number of patients with atypical bacterial respiratory infection = 12, number of patients with viral respiratory infection = 104)

Top right panel: Respiratory syncytial virus, RSV (number of patients with bacterial respiratory infection = 112, number of patients with RSV infection = 17).

Bottom left panel: Influenza virus (number of patients with bacterial respiratory infection = 112, number of patients with influenza infection = 27)

Bottom right panel: adenovirus (number of patients with bacterial respiratory infection = 112, number of patients with adenovirus = 17)*.*

Error bars represent 95% confidence interval. The host-protein signature assigned equivocal results to 10.3%, 11.6%, 11.5% and 14% of patients for the atypical bacteria, RSV, influenza and adenovirus subgroups, respectively. See Supplementary Figure
